# Selenized Polymer-Lipid Hybrid Nanoparticles for Oral Delivery of Tripterine with Ameliorative Oral Anti-Enteritis Activity and Bioavailability

**DOI:** 10.3390/pharmaceutics15030821

**Published:** 2023-03-02

**Authors:** Yuehong Ren, Chunli Qi, Shuxian Ruan, Guangshang Cao, Zhiguo Ma, Xingwang Zhang

**Affiliations:** 1 Department of Pharmaceutics, School of Pharmacy, Jinan University, No. 855 East Xingye Avenue, Guangzhou 511443, China; 2 Institute of Laboratory Animals, Jinan University, 601 West Huangpu Avenue, Guangzhou 510632, China; 3 Department of Pharmaceutics, Affiliated Hospital of Shandong University of Traditional Chinese Medicine, No. 16369 Jingshi Road, Jinan 250014, China

**Keywords:** tripterine, selenium, polymer-lipid hybrid nanoparticles, bioavailability, enteritis, anti-inflammation

## Abstract

The oral delivery of insoluble and enterotoxic drugs has been largely plagued by gastrointestinal irritation, side effects, and limited bioavailability. Tripterine (Tri) ranks as the hotspot of anti-inflammatory research other than inferior water-solubility and biocompatibility. This study was intended to develop selenized polymer-lipid hybrid nanoparticles loading Tri (Se@Tri-PLNs) for enteritis intervention by improving its cellular uptake and bioavailability. Se@Tri-PLNs were fabricated by a solvent diffusion-in situ reduction technique and characterized by particle size, ζ potential, morphology, and entrapment efficiency (*EE*). The cytotoxicity, cellular uptake, oral pharmacokinetics, and in vivo anti-inflammatory effect were evaluated. The resultant Se@Tri-PLNs were 123 nm around in particle size, with a PDI of 0.183, ζ potential of −29.70 mV, and *EE* of 98.95%. Se@Tri-PLNs exhibited retardant drug release and better stability in the digestive fluids compared with the unmodified counterpart (Tri-PLNs). Moreover, Se@Tri-PLNs manifested higher cellular uptake in Caco-2 cells as evidenced by flow cytometry and confocal microscopy. The oral bioavailability of Tri-PLNs and Se@Tri-PLNs was up to 280% and 397% relative to Tri suspensions, respectively. Furthermore, Se@Tri-PLNs demonstrated more potent in vivo anti-enteritis activity, which resulted in a marked resolution of ulcerative colitis. Polymer-lipid hybrid nanoparticles (PLNs) enabled drug supersaturation in the gut and the sustained release of Tri to facilitate absorption, while selenium surface engineering reinforced the formulation performance and in vivo anti-inflammatory efficacy. The present work provides a proof-of-concept for the combined therapy of inflammatory bowel disease (IBD) using phytomedicine and Se in an integrated nanosystem. Selenized PLNs loading anti-inflammatory phytomedicine may be valuable for the treatment of intractable inflammatory diseases.

## 1. Introduction

Inflammatory bowel disease (IBD) is a group of disorders that cause chronic inflammation (pain and swelling) in the intestine, mainly including Crohn’s disease (CD) and ulcerative colitis (UC) [1]. The incidence and prevalence of IBD are increasing worldwide year by year. It can affect people of all ages, from children to the aged, and affects all aspects of life. A clinical survey disclosed that the risk of colon cancer in IBD patients is higher than that in non-IBD patients [2]. Currently, the pathogenesis of IBD is still unclear. The basic options for treatment include surgery [3], medication [4], dietary intervention [5], and biologic therapy [6]. However, these medical strategies come with some downsides, such as a long course of treatment, side effects, and high recurrence. Therefore, it is important to develop novel medications for IBD therapy.

For the past few years, phytochemicals or phytocomponents have aroused considerable interest as therapeutic candidates in confronting various chronic diseases by virtue of their pleiotropic bioactivity and lower toxicity. Triperine (Tri) is a typical phytomedicine that has demonstrated good results in preclinical trials to treat diverse immune diseases, including IBD and rheumatoid arthritis (RA) [7]. It has been reported that Tri can upregulate protective autophagy through the PI3K/Akt/mTOR signaling pathway to improve experimental colitis in IL-10 deficient mice [8]. It can also ameliorate DSS-induced ulcerative colitis by modulating oxidative stress, the expression of inflammatory cytokines, and intestinal homeostasis [9]. These experimental studies indicate that Tri has a broad therapeutic prospect for IBD. However, its clinical translation is highly challenged by its lower aqueous solubility, poor oral bioavailability, and potential cytotoxicity. The water-solubility of Tri is merely 13.25 μg/mL around at 37 °C [10], but the oil-water partition coefficient (Log*P*) is as high as 5.63 [11], showing BCS IV-type drug properties.

To address the formulation challenge of Tri, a variety of pharmaceutical nanotechnologies have emerged, including polymerized prodrug micelles [12], enzyme-responsive nanoparticles [13], and bio-mimicking nanoparticles [14]. Polymer-lipid hybrid nanoparticles (PLNs) are next-generation core-shell nanostructures derived from liposome and polymeric nanoparticles, where a polymer core remains encysted by a lipid corona [15]. It relies on the lipid components to improve the intestinal permeability and biocompatibility of nanocarriers. The polymers help enhance drug encapsulation and gastrointestinal stability and take charge of the sustained release. PLNs have exhibited enormous potential in improving the physicochemical properties of drugs, overcoming the biological barriers, modulating drug release, and improving oral bioavailability [16]. Selenium (Se), as an essential trace element and physiological regulator, plays an important role in inflammation and immunity [17]. Previous studies have shown that Se functionalization can not only stabilize nanocarriers to facilitate GI transport, but also potentiate the curative effect of payloads through a synergistic mechanism [18,19,20,21,22,23]. Surface engineering with Se may be extra contributory to PLNs to maximize the antiphlogistic action of Tri.

In this study, selenized PLNs (Se@PLNs) were tactfully developed for the oral delivery of Tri with the intention of ameliorating its bioavailability and anti-enteritis activity. Tri-loaded Se@PLNs (Se@Tri-PLNs) were characterized by particle size, ζ potential, morphology, drug entrapment and release, and in vitro/vivo stability. The cellular uptake and transport feature of Se@Tri-PLNs were evaluated in Caco-2 cells. In addition, the in vivo pharmacokinetics and pharmacodynamics of Se@Tri-PLNs were demonstrated in normal rats and a DSS-induced murine ulcerative colitis (UC) model via oral dosing, respectively.

## 2. Materials and Methods

### 2.1. Materials

Tripterine was purchased from Anhui Zesheng Technology Co., Ltd. (Hefei, China). Soybean lecithin with phosphatidylcholine (PC) over 90% was obtained from Alfa Aesar Chemicals Co. Ltd. (Shanghai, China). Poly (lactic-co-glycolic acid) (PLGA 75:25) was provided by Shanghai Yuanye Bio-Technology Co., Ltd. (Shanghai, China). Sodium selenite (Na_2_SeO_3_) and reduced glutathione (r-GSH) were bought from Aladdin Reagent (Shanghai, China). Dulbecco’s Modified Eagle’s Minimal Essential Medium (DMEM), fetal bovine serum (FBS), and penicillin-streptomycin solution were purchased from Gibco BRL (Carlsbad, CA, USA). 3,3′-diocta-decyloxacarbocyanine perchlorate (DiO) and Hoechst 33258 were products of Shanghai Macklin Biochemical Co., Ltd. (Shanghai, China). Chlorpromazine, simvastatin, and genistein were obtained from Sigma–Aldrich (Shanghai, China). Dextran sodium sulfate (DSS) came from Coolaber Science and Technology Co., Ltd. (Beijing, China). Acetonitrile and methanol were provided by Merck (Darmstadt, Germany). All other chemicals were of analytical grade and used as received.

### 2.2. Cell Line and Animals

The Caco-2 cell line was obtained from ATCC (American Type Culture Collection). The cells were maintained in DMEM supplemented with 10% FBS and 100 U/mL penicillin and 100 μg/mL streptomycin at 37 °C in a humidified incubator of 5% CO_2_ and sub-cultured every 2–3 days.

Male Sprague Dawley (SD) rats (220 ± 20 g) and male BALB/c mice (18–22 g) were purchased from the Guangdong Medical Laboratory Animal Center (Guangzhou, China). All animals were housed under controlled conditions (23 ± 2 °C, 55 ± 10% humidity, and 12 h dark/light cycle) and free access to a standard laboratory diet and water. All mice were acclimatized for one week prior to the study. All of the protocols of animal experiments were reviewed and approved by the Experimental Animal Ethical Committee of Jinan University (No. 20220812-01). Animals were handled as per the Guidelines on the Care and Use of Animals for Scientific Purposes (2004).

### 2.3. Preparation of Se@Tri-PLNs

**Figure 1:**
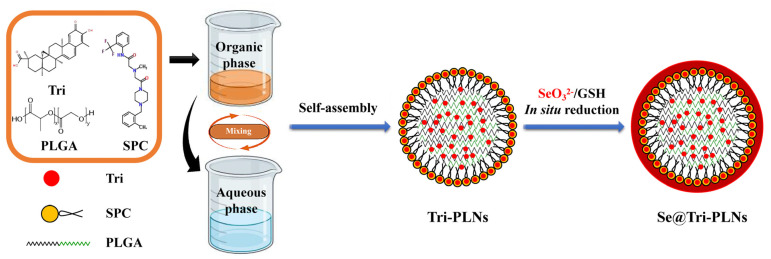
Schematic illustration of the structure and fabrication of Se@Tri-PLNs.

Se@Tri-PLNs were fabricated by solvent diffusion, followed by the in situ reduction technique, as shown in Figure 1 [19]. Briefly, Tri, lecithin and PLGA at a fixed ratio were weighed into a small flask, where a certain amount of binary organic solvent (acetone-ethanol, 4:1) was used to facilitate their dissolution to form an organic phase under ultrasonication. The organic solution was then dripped into deionized water dropwise in a fixed proportion under stirring at 1500 rpm for 0.5 h, resulting in the self-assembly of drug molecules and carrier materials into hybrid nanoparticles. Afterwards, the resultant nanoparticles were subjected to sonication for 6 min. After removal of organic solvents via evaporation, Tri-loaded polymer-lipid hybrid nanoparticles (Tri-PLNs) were obtained. Subsequently, Na_2_SeO_3_ was introduced into the nanosuspensions of Tri-PLNs and stirred for 0.5 h at room temperature. After that, r-GSH was added into the system to trigger the reduction reaction at a molar ratio of 4:1 to Na_2_SeO_3_. The reaction was maintained for 2.5 h at 37 °C under an agitation of 1000 rpm. Se@Tri-PLNs were formed upon Se^4+^ being reduced into elemental Se and precipitating onto the surface of Tri-PLNs. The residual reactants were dialyzed out against deionized water twice. To obtain a preferred formulation, we screened the factors affecting the formulation performance of Se@Tri-PLNs, including the mass ratio of lecithin to PLGA, the mass ratio of Tri to carrier materials, the volume ratio of organic phase to the water phase upon self-assembly, and the concentration of Na_2_SeO_3_ upon reaction.

### 2.4. Characterization of Nanocarriers

Tri-PLNs and Se@Tri-PLNs were characterized by particle size, ζ potential, morphology, entrapment efficiency (*EE*), and drug loading (*DL*). The particle size and ζ potential of nanoparticles were measured by size and potential analyzer (Zetasizer Nano ZS, Malvern, UK) at 25 °C after dilution with deionized water approximately 50 times in a quartz cell, based on dynamic light scattering and Doppler velocimetry, respectively. The data were obtained using the built-in software for analysis of particle size and ζ potential.

To inspect the morphology of nanocarriers, the samples were diluted properly with deionized water and fixed on a carbon-coated copper grid by evaporating the residual water naturally. Microimaging was performed on a JEM-1230 transmission electron microscope (TEM) (JEOL, Tokyo, Japan). The micrographs of Tri-PLNs and Se@Tri-PLNs were taken at an acceleration voltage of 100 kV.

The *EE* and *DL* of Tri-PLNs and Se@Tri-PLNs were determined after separating unentrapped and free Tri from nanosuspensions by the centrifugal ultrafiltration technique. Freshly prepared Tri-PLNs and Se@Tri-PLNs were first centrifuged at 5000 rpm for 10 min to remove the unentrapped coarse Tri, and then subjected to centrifugal ultrafiltration against a MWCO 30 K filter device (Amicon^®^, Merck, Darmstadt, Germany). The total drug and free drug in the filtrate were quantified by UPLC, as described below. *EE* and *DL* were calculated as follows:

$$EE(\%)=(1-\frac{M_{fre}}{M_{tot}})\times 100;\;DL(\%)=\frac{M_{tot}-M_{fre}}{\left(M_{tot}+M_{exc}\right)}\times 100$$

where *M_fre_*, *M_tot_* and *M_exc_* denote the amounts of free Tri, total Tri, and excipients used in the formulation, respectively.

A Waters ACQUITY H-Class ULPC system equipped with a quaternary pump, an autosampler, and a PDA detector was employed for the Tri assay. The samples were eluted against a Poroshell HPH-C_18_ column (2.6 μm, 2.1 × 50 mm, Waters) at 45 °C with 5 μL of injection. A mobile phase composed of methanol and 0.25% phosphoric acid solution (80/20) pumped at a flow rate of 0.2 mL/min was utilized to elute the samples. The elution signals were collected at 425 nm.

### 2.5. Biorelevant Stability Study

The stability of nanocarriers in the simulated gastric fluid (SGF) and simulated intestinal fluid (SIF) was evaluated. With deionized water medium as the control, the changes in particle size, PDI and ζ potential of Tri-PLNs and Se@Tri-PLNs in SGF and SIF were investigated, respectively. In detail, an appropriate amount of nanosuspensions was added into SGF (containing 0.32% pepsin, pH 1.2) or SIF (containing 1% trypsin, pH 6.8) at a volume of 1:5 and incubated at 37°C under stirring at 100 rpm. At predetermined intervals, 1 mL of sample was withdrawn and immediately analyzed for particle size, PDI and ζ potential. To get insight into the mechanism of particle size change, we reassessed the indices of particle size by changing the pH of the test medium that nanocarriers were susceptible to after incubation. The same sample was divided into two parts: one was directly determined for particle size, PDI and ζ potential after incubation, and the other was determined after neutralization with a pH conditioner.

### 2.6. In Vitro Release Study

The in vitro release of Tri-PLNs and Se@Tri-PLNs were conducted in deionized water, pH 1.2 HCl solution and pH 6.8 phosphate buffered saline (PBS) containing 0.5% Tween 80 as a solubilizing agent, respectively. Briefly, 2.5 mL of Tri-PLNs or Se@Tri-PLNs were placed into dialysis bags loading 50 mL of release medium and subjected to agitation at 100 rpm and 37 °C for 12 h in the dissolution cups using a ZRS-8GD dissolution tester (TIANDA TIANFA, Tianjing, China). At 0.5, 1, 2, 4, 6, 8 and 12 h, 1 mL of release solution was withdrawn and filtered against a 0.22 μM filter membrane. Tri concentration in the filtrates was analyzed by UPLC, as described above. The release profiles were plotted according to the accumulative release percentage with the time.

### 2.7. Cytotoxicity Assay

The cytotoxicity of Tri, Tri-PLNs and Se@Tri-PLNs was evaluated by the MTT method. Briefly, Caco-2 cells were cultured in DMEM medium supplemented with 10% inactivated FBS and 1% dual antibiotic solution (100 U/mL of penicillin-100 μg/mL of streptomycin) at 37 °C under a 5% CO_2_/95% air atmosphere. Caco-2 cells were seeded into a 96-well plate and cultured over 24 h. The cell viability was evaluated by colorimetry using a Synergy H1 Microplate Reader (BioTek, Winooski, VT, USA) after incubation for 8 h, 12 h and 24 h with a series of concentrations of Tri. After finalizing the test concentration and time, the cell viability was assessed again in the presence of Tri, Tri-PLNs, Se@Tri-PLNs, or blank carrier (Se@PLNs).

### 2.8. Cellular Uptake and Internalization

Cellular uptake and internalization of Tri-PLNs and Se@Tri-PLNs were analyzed by flow cytometry and CLSM imaging, respectively [20]. DiO-labeled Tri-PLNs and Se@Tri-PLNs were freshly prepared and utilized for observation of cellular uptake and internalization. Except for light avoidance, the preparation of DiO-labeled Tri-PLNs and Se@Tri-PLNs followed the same procedures of Tri-PLNs and Se@Tri-PLNs by synchronously dissolving DiO into the organic phase upon fabrication. Fluorescent Tri-PLNs and Se@Tri-PLNs were incubated with Caco-2 cells at a Tri concentration of 5 μg/mL for 0.5, 1, and 2 h at 37 °C, respectively. The cells were then rinsed twice with a pH 7.4 HBSS and the uptake rate was examined using a flow cytometer (FACSCanto, BD, New York, NY, USA).

To identify the cellular internalization, Caco-2 cells were seeded in a confocal Petri dish that was balanced with a cell culture medium in advance. Cells were cultured for 24 h at a density of 5 × 10^4^ cell/mL. DiO-labeled Tri-PLNs and Se@Tri-PLNs were then introduced into the confocal dish to incubate for 1 h. After incubation, the cells were washed two times with cold HBSS, fixed in 4% paraformaldehyde for 0.5 h, and stained with Hoechst 33258. The internalized nanoparticles by Caco-2 cells were visualized on a LSM800 confocal laser scanning microscope (CLSM, Zeiss, Wetzlar, Germany).

### 2.9. Cellular Trafficking Pathway

The fluorescent nanoparticles prepared above were used to illustrate the cellular trafficking pathway of Tri-PLNs and Se@Tri-PLNs using various transmembrane transport inhibitors for intervention [24]. Caco-2 cells were pretreated with inhibitors at 37 °C or treated at 4 °C for 0.5 h. DiO-labeled Tri-PLNs and Se@Tri-PLNs were then introduced into the cell wells and incubated for another 2 h. Thereafter, the cells were washed, trypsinized with trypsin-EDTA (0.25%) solution, and centrifuged at 1000 rpm to collect cell pellets. The fluorescence intensity in the sample of cell pellets was quantified by flow cytometry. The internalization or endocytosis pathway of nanoparticles was deciphered according to the altered cellular uptake in the presence of various inhibitors. The concentration and function of physiological inhibitors used are listed in Table 1.

### 2.10. Oral Pharmacokinetics

Male SD rats were fasted overnight prior to administration, but were allowed free access to water. The rats were randomized into three groups (*n* = 5) and orally administered with Tri suspensions, Tri-PLNs or Se@Tri-PLNs at a dose of 40 mg/kg, respectively. At the time points of 0.25, 0.5, 1, 2, 4, 6, 8, 12 and 24 h, aliquots of blood (~0.25 mL) were sampled from the caudal vein. Fresh plasma was immediately prepared by centrifugation at 3000 rpm for 10 min, during which the concentration of Tri was measured by an UPLC-MS method, as described below. The blood drug concentration versus time curve was plotted, and pharmacokinetic parameters were analyzed by *PK*Solver 2.0.

To quantify the plasma Tri, a liquid-liquid extraction procedure was used to extract Tri from the plasma [25]. Briefly, 100 mL of plasma were combined with 500 mL of ethyl acetate supplemented with 0.1 μg of emodin as an internal standard and then centrifuged to separate the supernatant. The plasma was extracted twice, and the extracting solution was merged followed by evaporation at 30 °C under a vacuum. The residues were reconstituted in 100 μL of methanol for UPLC-MS analysis (SCIEX Triple Quad LC-MS/MS system, AB SCIEX, Framingham, MA, USA). The samples were eluted against a Poroshell HPH-C_18_ column (2.6 μm, 2.1 × 50 mm, Waters) at 35 °C with 5 μL of injection. A gradient elution was applied using 1% formic acid in water (mobile phase A) versus 0.1% formic acid in acetonitrile (mobile phase B) at a flow rate of 0.4 mL/min. The gradient elution program was 60% B at 0–1 min, 68% B at 1–5 min, 100% B at 5–9.5 min, and 60% B at 9.4–12 min.

### 2.11. In Vivo Anti-Enteritis Activity Evaluation

A mouse UC model was adopted to evaluate the in vivo anti-inflammatory activity of Se@Tri-PLNs. UC was induced by DSS in the drinking water (3%, *w*/*v*) for one week to establish the UC model. In an experiment, mice were randomly divided into five groups, i.e., control, DSS (model), DSS + Tri suspensions, DSS + Tri-PLNs, and DSS + Se@Tri-PLNs (*n* = 5). The mice in the control group were provided with normal drinking water, while all mice in other groups were given water containing 3% DSS for seven days. From the fourth day, the mice in the treatment group were orally administered with Tri suspensions, either Tri-PLNs or Se@Tri-PLNs by gavage at a dose of 1 mg/kg every day for four consecutive days. The mice were monitored for body weight, disease activity index (DAI), and colon morphology. Weight change, fecal viscosity and occult blood were collectively scored from 0 to 4 to calculate DAI according to the previous report [26]. On the 11th day, the mice were euthanized, and the serum, thymus, spleen, and colon tissues were collected immediately. All serum and colon samples were stored at −80 °C until analysis.

A histological examination was performed on the colon tissue by blinded analysis. The severity of colon damage was checked by routine H.E. staining. The levels of TNF-α, IL-1β and IL-6 in the serum were measured using commercial mouse TNF-α, IL-1β, and IL-6 ELISA kits (Andygene, Beijing, China), respectively. The thymus and spleen were weighed under sterile conditions and used to calculate the thymus index [(thymus weight (mg)/mouse weight (g)) × 10] and spleen index [(spleen weight (mg)/mouse weight (g)) × 10].

## 3. Results and Discussion

### 3.1. Preparation and Characterization of Se@Tri-PLNs

**Figure 2:**
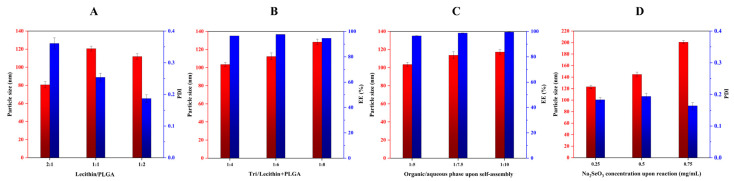
Formulation factors affecting the particle size, polydispersity index (PDI), and entrapment efficiency (*EE*) of nanoparticles: (**A**) mass ratio of lecithin to PLGA; (**B**) mass ratio of Tri/carrier materials (lecithin + PLGA); (**C**) phase ratio of organic to aqueous (*v*/*v*); and (**D**) Na_2_SeO_3_ concentration upon in situ reduction.

Solvent diffusion or the nanoprecipitation technique is the most common method for preparing matrix nanoparticles [27,28,29]. When water-miscible organic solvents diffuse towards the aqueous phase, self-assembly that forms nanoparticles takes place due to the sharp decline in the solubility of materials. In the preliminary study, we found that the ratios of lecithin/PLGA, drug/carrier materials, organic/aqueous phase upon self-assembly, and the concentration of Na_2_SeO_3_ upon selenization affected the formulation performance. Figure 2 shows the effects of these formulation variables on the particle size and PDI or *EE* of PLNs. It was observed that the mass ratio of lecithin to PLGA exhibited a peculiar effect on the particle size and PDI of Tri-PLNs (Figure 2A). The high ratio of lecithin resulted in smaller nanoparticles, but they were broadly dispersed. This may be related to the formation of heterogeneous nanoparticles, such as vesicles and micelles, in addition to PLNs. The formulation with a mass ratio of lecithin/PLGA at 1:2 produced the smallest nanoparticles with the narrowest polydispersity. Therefore, it could be argued that lecithin only played a surfactant role in the formulation to facilitate the formation of PLNs as a lipid corona, rather than as the principal carrier material. As the lecithin/PLGA ratio fixed, the ratios of drug/carrier materials and the organic/aqueous phase upon self-assembly had negative effects on the particle size of Tri-PLNs (Figure 2B,C). They increased with the increase of the ratios of Tri in the formulation and aqueous phase upon preparation. However, there was no significant difference in *EE*, which may be attributed to the high lipophilicity of Tri [30]. Interestingly, the particle size of Se@Tri-PLNs was significantly increased with the increased concentration of Na_2_SeO_3_ upon in situ reduction (Figure 2D). This phenomenon illustrated that the nascent elemental selenium had successfully precipitated on the surface of Tri-PLNs. The concentration of Na_2_SeO_3_ at 0.25 mg/mL was found to be optimal for decorating Tri-PLNs to fabricate Se@Tri-PLNs with a suitable particle size. 

**Figure 3:**
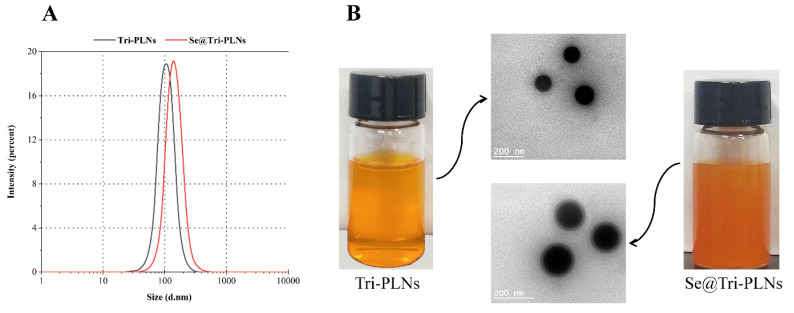
Characterization of Tri-PLNs and Se@Tri-PLNs by particle size distribution (**A**) and appearance/TEM micrographs (**B**).

Taking the above together, the formulation and process of Se@Tri-PLNs were finalized as lecithin to PLGA at a ratio of 1:2, Tri to lecithin and PLGA at a ratio of 1:4, the organic phase to the water phase at a volume ratio of 1:5 upon self-assembly, and 0.25 mg/mL of Na_2_SeO_3_ upon in situ reduction. Based on the preferred formulation and process, Se@Tri-PLNs were prepared using 3 mg of Tri, 4 mg of SPC, and 8 mg of PLGA. Drug and carrier materials were dissolved in 3 mL of an acetone-ethanol binary organic system that was then dripped into 15 mL of water followed by in situ reduction with 3.75 mg of Na_2_SeO_3_ and a quadruple mole of r-GSH. The resultant Se@Tri-PLNs possessed a particle size of 123.1 nm around with a PDI of 0.183 (Figure 3A). The particle size was larger than that of unmodified Tri-PLNs (103.5 nm). The increase in particle size can be attributable to the Se precipitation onto the surface of Tri-PLNs. The nanosuspensions of Se@Tri-PLNs were reddish-brown in appearance, which differed from that of Tri-PLNs (yellow) due to the red Se attachment (Figure 3B). The ζ potential of nanoparticles changed from −39.40 mV to −29.70 mV before and after selenization. The absolute ζ potentials were all greater than 25 mV, suggesting good colloidal stability for them. Both Tri-PLNs and Se@Tri-PLNs were spherical in morphology, as revealed by TEM. Otherwise, Se@Tri-PLNs exhibited an apparent corona around the nanoparticles, manifesting the occurrence of Se precipitation. The *EE* of Se@Tri-PLNs was as high as 98.95%, with a *DL* of 17.07%. The merits of small particle size and high encapsulation with Se@Tri-PLNs create conditions for synergy and attenuation of Tri after oral administration.

### 3.2. Gastrointestinal Stability

**Figure 4:**
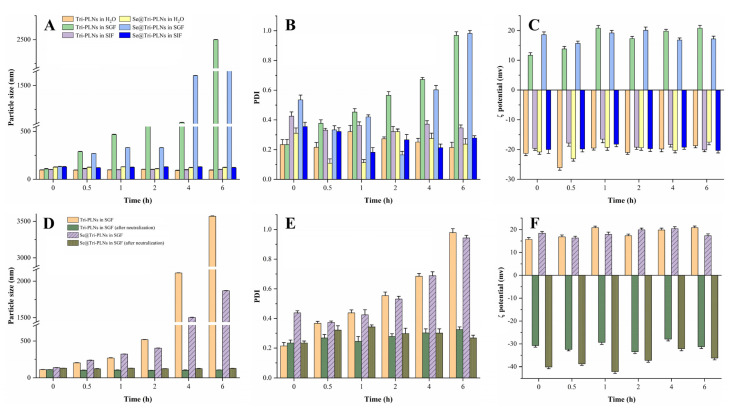
Stability of Tri-PLNs and Se@Tri-PLNs in biorelevant fluids: (**A**–**C**) changes in particle size, PDI and ζ potential of Tri-PLNs and Se@Tri-PLNs as incubated with deionized water, SGF and SIF for different times; (**D**–**F**) changes in particle size, PDI and ζ potential of Tri-PLNs and Se@Tri-PLNs in SGF before and after pH neutralization with sodium hydroxide (*n* = 3, mean ± SD).

The in vivo fate of nanocarriers is closely associated with their absorption and drug delivery efficacy. The physicochemical stability of Tri-PLNs and Se@Tri-PLNs can be manifested by the particle size, PDI and ζ potential. Biorelevant media including deionized water, SGF and SIF were applied for evaluating the GI stability of Tri-PLNs and Se@Tri-PLNs. Figure 4 shows the changes in particle size, PDI and ζ potential of two nanocarriers with time upon incubation with deionized water, SGF and SIF. It turned out that Tri-PLNs and Se@Tri-PLNs were rather stable both in deionized water and SIF, except in SGF, with no significant changes in particle size, PDI and ζ potential (Figure 4A–C). However, in the case of SGF, these indicators changed dramatically, whereby the particle size increased significantly along with the rise of PDI and the inversion of interfacial charge. The alterations were more pronounced in terms of Tri-PLNs. In order to elucidate the underlying cause, we conducted pH neutralization to the samples incubated with SGF and determined the parameters again. After being neutralized to pH 7.4 with sodium hydroxide, the particle size, PDI and ζ potential of Tri-PLNs and Se@Tri-PLNs was restored to almost their initial levels. The results indicated that Tri-PLNs and Se@Tri-PLNs were not digested or degraded in the harsh gastric condition, but rather protonated. Both Tri-PLNs and Se@Tri-PLNs were negatively charged on the surface, and thus they tended to appear protonated in the strong acidic environment. Protonation tends to cause the aggregation of nanoparticles, the enlargement of PDI, and the reversal of interfacial charge [31]. Of note, Se@Tri-PLNs exhibited a stronger resistance to acid protonation compared with Tri-PLNs, owing to surface Se attachment.

### 3.3. In Vitro Drug Release

**Figure 5:**
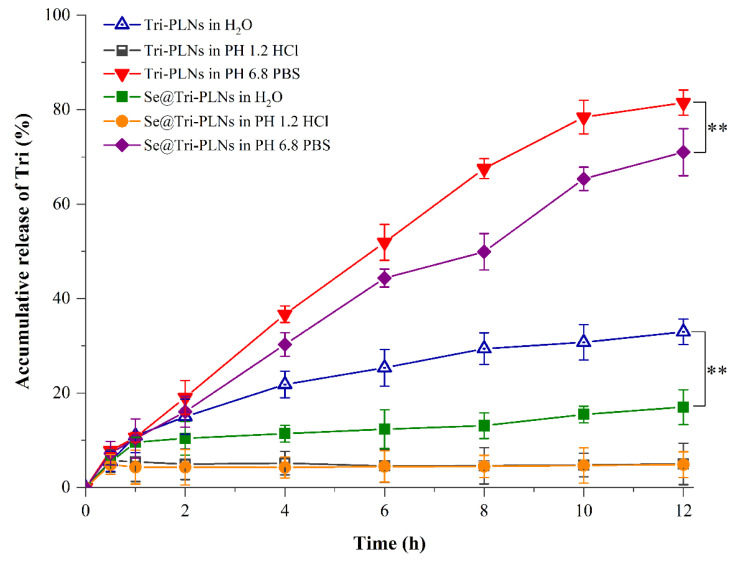
Release profiles of Tri from Tri-PLNs and Se@Tri-PLNs in deionized water, pH 1.2 HCl solution, and pH 6.8 phosphate buffer solution (PBS). Data expressed as mean ± SD (*n* = 3), paired *t*-test, ** *p* < 0.01.

The release profiles of Tri from Tri-PLNs and Se@Tri-PLNs in different media are delimitated in Figure 5. In pH 1.2 HCl solution, both Tri-PLNs and Se@Tri-PLNs exhibited an extremely low release, where merely 5.02% and 4.89% of Tri were released from Tri-PLNs and Se@Tri-PLNs within 12 h, respectively. As suggested in the stability study, the marginal release of Tri from nanoparticles may result from protonation of the surface of the nanoparticles. Protonation causes nanoparticle aggregation and contraction that retards drug release. Drug release was accelerated in deionized water. Nevertheless, the accumulative release percentages were still limited for both Tri-PLNs and Se@Tri-PLNs. The maximal release percentage was not more than 38% within the predictable time of GI transportation, showing an obvious sustained release effect. Quicker drug release was presented in the medium of pH 6.8 PBS. This may be related to the ionization of Tri in the buffering system. Tri contains a structural carboxyl that can react with alkali ions such as Na^+^ and K^+^ to form salts, thus promoting the dissolution of Tri from PLNs. In comparison, Se@Tri-PLNs displayed slower drug release both in deionized water and pH 6.8 PBS. The accumulative release rate of Tri exceeded 81.50% within 12 h in the case of Tri-PLNs in the medium of pH 6.8 PBS, whereas it was 71.03% with regard to Se@Tri-PLNs. The reduced release indicated that a thin Se layer was provided with Se@Tri-PLNs as a result of in situ reduction. The characteristics of pH-dependent drug release reduces the premature release of Tri in the stomach, which will be conducive to its intestinal absorption through integral nanoparticles, hence improving the oral bioavailability.

### 3.4. Cytotoxicity

**Figure 6:**
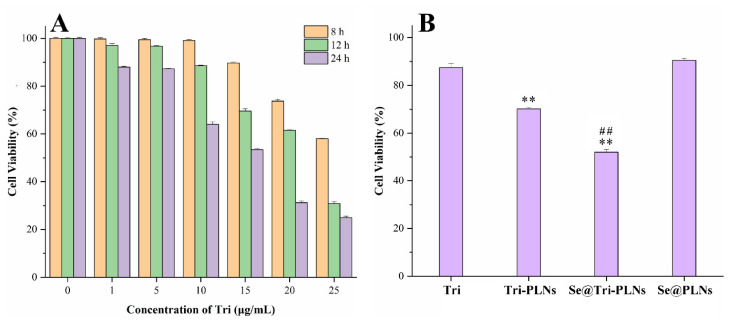
Cell viability analysis of free and formulated Tri: (**A**) cell viability after incubation with different concentrations of free Tri for 8 h, 12 h and 24 h; (**B**) cell viability of formulated Tri (Tri-PLNs and Se@Tri-PLNs) for 24 h at a concentration of 5 μg/mL with free Tri and blank nanoparticles (Se@PLNs) as references. Data expressed as mean ± SD (*n* = 6), ANOVA, ** *p* < 0.01, compared with Tri, ^##^ *p*< 0.01, compared with Tri-PLNs.

Figure 6 depicts the cytotoxicity of free Tri, formulated Tri and a blank carrier in Caco-2 cells. As shown in Figure 6A, the cytotoxicity of Tri was time- and concentration-dependent. When the concentration of Tri was 1–5 μg/mL, the cell survival rate was higher than 80% over 24 h. Above this concentration, the cytotoxicity of Tri became apparent (Figure 6A). To this end, it is of interest to formulate Tri into nanomedicine so as to overcome the issues of low solubility and limited oral absorption due to drug efflux [21,32]. To further examine the cytotoxicity of nanoscaled Tri (Tri-PLNs and Se@Tri-PLNs), the cell viability was measured at a given concentration equivalent to 5 μg/mL of Tri after incubation for 24 h. A blank carrier (Se@PLNs) exhibited lower cytotoxicity parallel to free Tri. However, Tri-PLNs and Se@Tri-PLNs produced a certain cytotoxicity to Caco-2 cells, where the cell viability was reduced to 58.36% and 52.01% after treatment for 24 h at the concentration of 5 μg/mL, respectively, which were significantly lower than that of free Tri at the same level. This can be explained by the increased cellular uptake of Tri-PLNs and Se@Tri-PLNs that enhances the intracellular concentration of Tri, which results in more cell necrosis. Tri has been proven to be a substrate of P-glycoprotein with significant intestinal efflux [33]. Thus, the increased cytotoxicity of formulated Tri results from incremental cellular uptake rather than the nanotoxicity from the carriers. In addition, there is no need to worry about damage to enterocytes caused by nanoparticles, since the concentration exposed to the intestinal epithelial cells will be much lower than the tested concentration after oral administration [34].

### 3.5. Cellular Uptake, Internalization, and Transport Mechanisms

**Figure 7:**
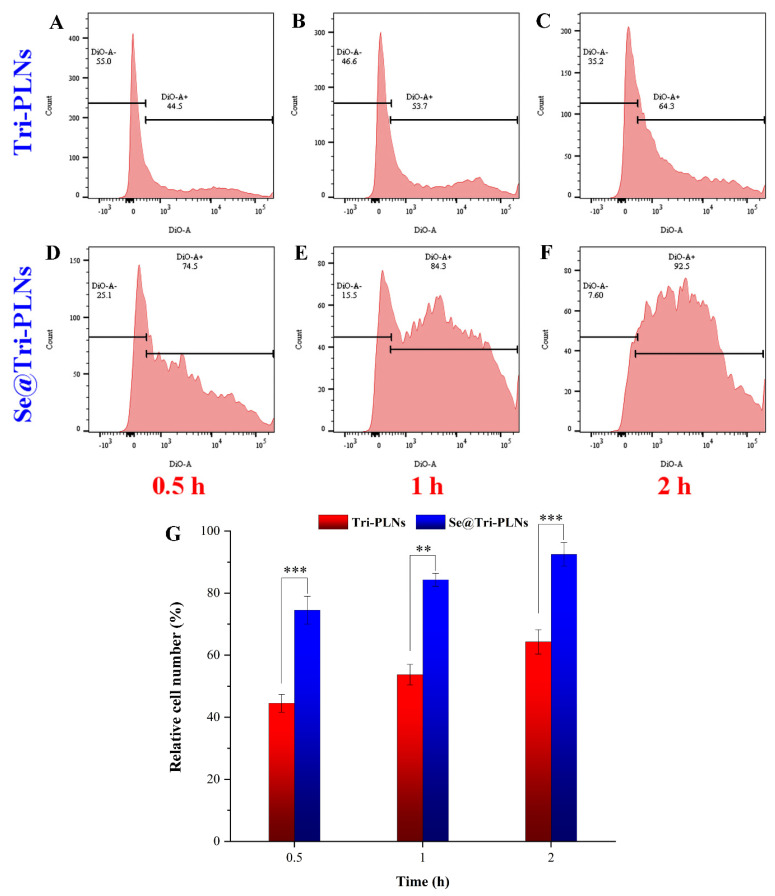
Cellular uptake of Tri-PLNs and Se@Tri-PLNs quantified by flow cytometry using fluorescently labeled nanoparticles after incubation with Caco-2 cells for 0.5, 1, and 2 h. (**A**–**F**): statistical distribution diagrams of positive cells; (**G**): histograms of relative positive cells. Data expressed as mean ± SD (*n* = 3), paired *t*-test, ** *p* < 0.01, *** *p* < 0.001.

The in vitro cellular uptake of nanoparticles tested on enterocytes is normally used to predict the in vivo absorption thereof. Figure 7 shows the flow cytometric events of cellular uptake in Caco-2 cells with regard to Tri-PLNs and Se@Tri-PLNs. A time-dependent cellular uptake was presented by both nanocarriers. The relative cell numbers stained by DiO-labeled Tri-PLNs and Se@Tri-PLNs were as high as 64.3% and 92.5%, respectively, after incubation for 2 h. By contrast, the cellular uptake rate of Se@Tri-PLNs was higher. It was reported that this was a result of the heavy density of selenized nanocarriers, which resulted in enhancive nonspecific phagocytosis [22]. Oral vehicles that we developed produced a similar cellular uptake to cationic liposomes layered by trimethylated chitosan [35], showing stronger intestinal epithelial permeability.

**Figure 8:**
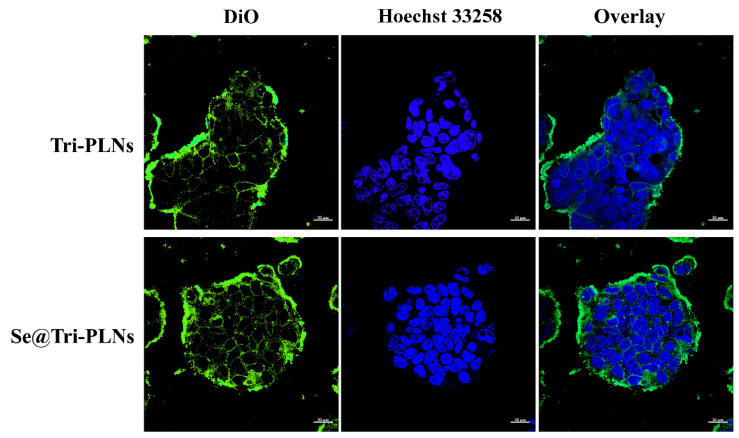
Cellular internalization of Tri-PLNs and Se@Tri-PLNs characterized by CLSM after incubation with Caco-2 cells for 1 h. Scale bar: 20 μm.

The excellent cell affinity of Tri-PLNs and Se@Tri-PLNs can also be reflected by CLSM imaging of cellular internalization. The apparent fluorescence staining associated with Tri-PLNs and Se@Tri-PLNs was observed to diffusely distribute in the cell colony (Figure 8). The fluorescence intensity in the group of Se@Tri-PLNs was slightly stronger than that of Tri-PLNs, consistent with the results of cellular uptake. The findings suggest that selenized nanoparticles take on a certain absorption-promoting effect which may contribute to the enhancement of oral bioavailability. Furthermore, it could be seen from the micrograph that nanoparticle-associated fluorescence mainly distributed within the cytoplasm rather than the nucleus (stained by Hoechst 33258). Therefore, it is fairly beneficial to the translocation of nanoparticles from the apical membrane of intestinal epithelium to the basolateral side.

**Figure 9:**
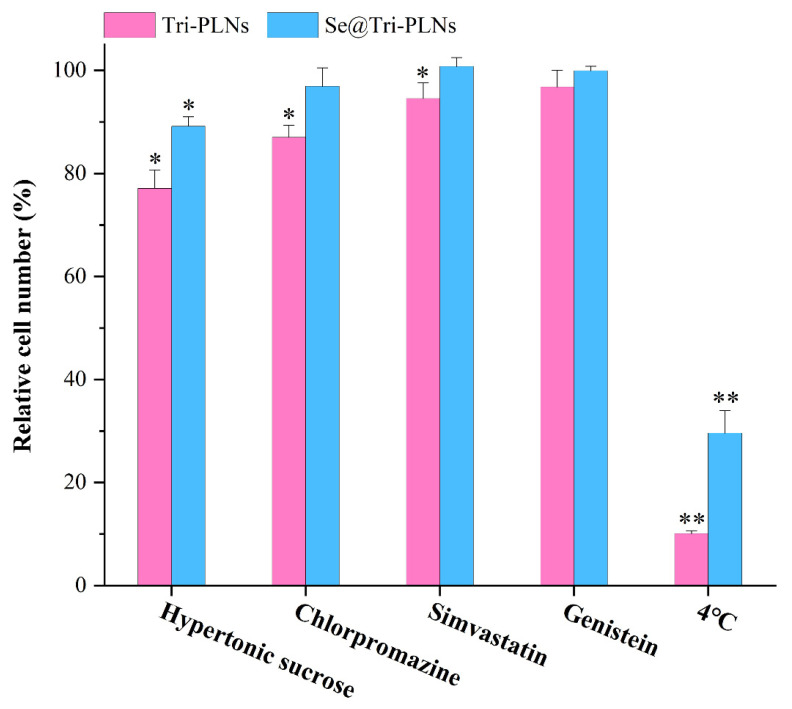
Cellular trafficking pathway of Tri-PLNs and Se@Tri-PLNs interpreted by relative cellular uptake in the presence of various physiological inhibitors or treated under 4 °C (*n* = 3). Hypertonic sucrose and chlorpromazine act as nonspecific and specific clathrin-mediated endocytosis inhibitors, respectively; simvastatin and genistein serve as nonspecific and specific caveolin-mediated endocytosis inhibitors, respectively. ANOVA, * *p* < 0.05, ** *p* < 0.01, compared with control.

The cellular uptake of Tri-PLNs and Se@Tri-PLNs in the presence of various inhibitors treated at 4 °C is shown in Figure 9. The cellular uptake rate of Tri-PLNs was significantly affected by hypertonic sucrose, chlorpromazine, and simvastatin, whereas genistein exerted less effect on the cellular uptake. Hypertonic sucrose markedly inhibited the uptake of Se@Tri-PLNs, with a reduction of 10.83% in the uptake rate compared with the control group. The effects of chlorpromazine, simvastatin and genistein on the cellular uptake of Se@Tri-PLNs were relatively insignificant. In addition, the cellular uptakes of Tri-PLNs and Se@Tri-PLNs were markedly exhibited at 4 °C, resulting in an uptake reduction of 89.3% and 70.6%, respectively. These results suggest that Tri-PLNs and Se@Tri-PLNs share different cellular uptake mechanisms. Clathrin-mediated endocytosis and nonspecific caveolin-mediated endocytosis are involved in the uptake process of Tri-PLNs, but ATP-dependent transport through a special transporter or pinocytosis may be mainly responsible for the cellular uptake of Se@Tri-PLNs. It is known that some transporters are sensitive to temperature, and thus active transport is greatly inhibited under a lower temperature [36]. However, the specific transporter responsible for the transport of Se@Tri-PLNs is not clear. Nevertheless, the active transport manner enables Se@Tri-PLNs to be more readily assimilated by the absorptive intestinal epithelia.

### 3.6. Enhanced Bioavailability

**Figure 10:**
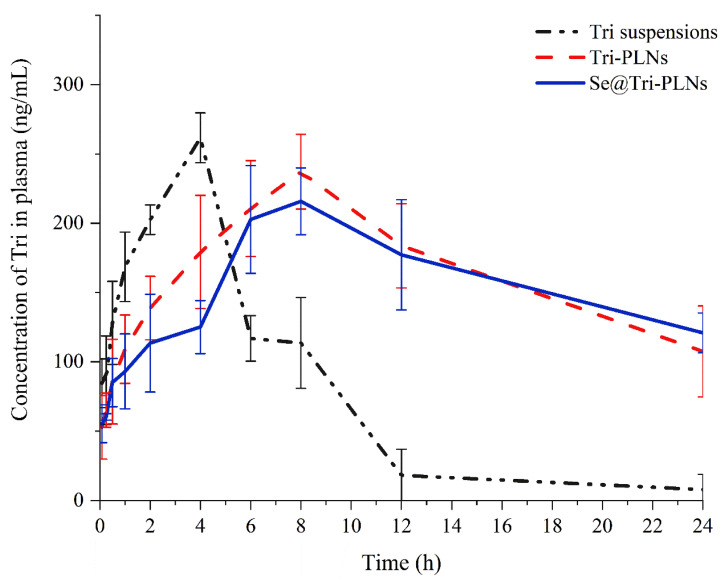
Plasma drug concentration vs. time profiles of Tri suspensions, Tri-PLNs and Se@Tri-PLNs in rats after oral administration with a dose of 40 mg/kg (mean ± SD, *n* = 5).

The pharmacokinetic profiles of Tri suspensions, Tri-PLNs and Se@Tri-PLNs in SD rats after oral administration are shown in Figure 10. It could be seen that the blood drug concentration in the group of Tri suspensions went up quickly but declined quickly as well, presenting a short in vivo residence of free Tri. As formulated into Tri-PLNs and Se@Tri-PLNs, the absorption extent of Tri was dramatically enhanced, though the rate of absorption decreased. The maximum plasma concentration (*C*_max_) did not drop much for Tri-PLNs, but declined a little for Se@Tri-PLNs. The time to *C*_max_ (*T*_max_) lagged significantly behind the suspension formulation, from approximately 4 h to 8 h (Table 2). Meanwhile, the mean residence time (*MRT*) of Tri-PLNs and Se@Tri-PLNs increased accordingly, increasing to approximately 13.8 h. Between Tri-PLNs and Se@Tri-PLNs, there was no significant difference in the absorption rate as signified by the parameters of *C*_max_ and *T*_max_. However, the degree of absorption was enhanced substantially in terms of Se@Tri-PLNs compared to Tri-PLNs. The high blood drug concentration was maintained, even at the end of sampling. These results suggest that Se@Tri-PLNs possesses a prolonged absorption profile, which shows good in vitro and in vivo correlation (IVIVC), combined with the in vitro release. The relative oral bioavailability of Tri-PLNs and Se@Tri-PLNs calculated by the trapezoidal method was up to 280% and 397% compared with the Tri suspensions, respectively. Furthermore, the oral bioavailability that resulted from Se@Tri-PLNs was greater than that of Tri-PLNs, just as Se@Tri-PLNs could better sustain the release of Tri. Sustained and everlasting absorption can maintain a long-term curative effect in vivo, which is beneficial for the treatment of chronic diseases [37].

To improve the bioavailability of Tri, a variety of oral nano-vehicles have been explored, including phytosomes [20,38], silk fibroin nanoparticles [39], and lactosylated albumin nanoparticles [40]. Compared with silk fibroin nanoparticles and lactosylated albumin nanoparticles, the selenized PLN system developed by us resulted in higher blood drug concentrations, in addition to longer in vivo residence times. The *C*_max_ that resulted from silk fibroin nanoparticles was only 90.5 ± 49.2 ng/mL, while this parameter of lactosylated albumin nanoparticles was lower than 3.5 ng/mL. Depending on the superior gastrointestinal stability, our constructed nano-vehicle exhibits an advantage in ameliorating the oral absorption of Tri.

### 3.7. Ameliorative In Vivo Anti-Enteritis

**Figure 11:**
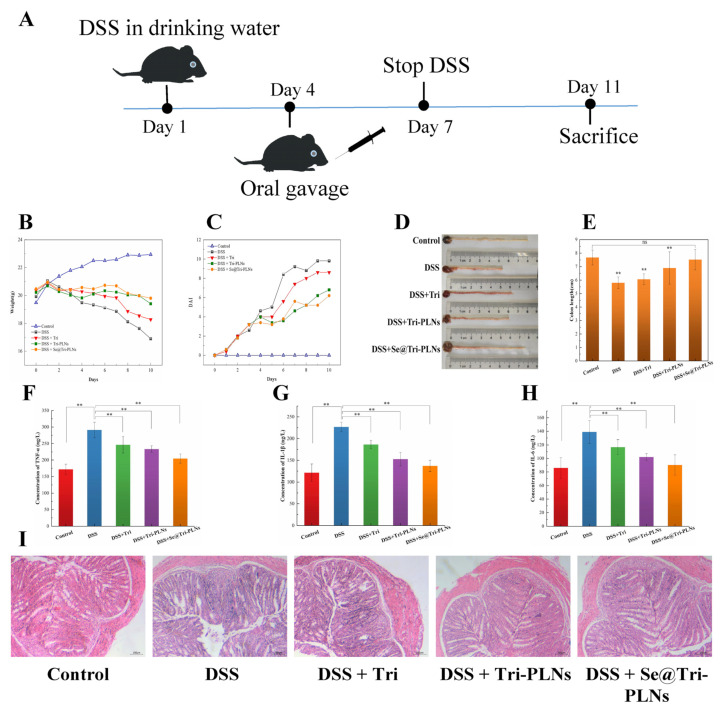
In vivo anti-inflammation evaluation based on a DSS-induced murine ulcerative colitis model: (**A**) schematic diagram of modeling and treatment; (**B**) changes in body weight; (**C**) DAI index; (**D**) colon imaging; (**E**) colon length; (**F**–**H**) serumal levels of TNF-α, IL-1β and IL-6; and (**I**) histopathological examination of the colon. Paired *t*-test, ** *p* < 0.01, ns: no statistical difference.

A murine UC model was adopted to evaluate the anti-enteritis effect of Se@Tri-PLNs. As shown in Figure 11B,D, the control mice exhibited unceasing weight gain and normal colon histology. However, the model mice emerged with significant weight loss along with rectal bleeding and diarrhea after DSS induction for three days. Meanwhile, the length of the colon of the model mice was shortened, and the colon tissue presented inflammatory symptoms, such as ulcers, crypt abscesses, loss of goblet cells and mucus layers, and considerable neutrophil infiltration into the lamina propria (Figure 11D,I). These results indicated that the UC model based on BALB/c mice was successfully established by the oral administration of 3% DSS in drinking water.

After treatment with Tri suspensions, either with Tri-PLNs or Se@Tri-PLNs for one week at the dose of 1 mg/kg, the mice in the trial groups had increased body weight, a decreased DAI score, elongated colon length, and alleviated colon injury to different extents in comparison with the model group (Figure 11B–E,I). It is clear that both free Tri and formulated Tri are provided with good anti-UC activities, among which the curative effect of Se@Tri-PLNs was most prominent. The results also demonstrated that Se could potentiate the anti-inflammatory action of Tri on the base of PLNs. The synergistic effect between Se and therapeutic molecules has been widely confirmed [34,41,42]. This was well supported by the declined levels of inflammatory factors in the peripheral blood (Figure 11F–H). The normal mice (control) maintained lower levels of serumal TNF-α, IL-1β, and IL-6, whereas three inflammatory factors were clearly upregulated in the DSS-induced group. After treatment with Tri suspensions, Tri-PLNs and Se@Tri-PLNs for one week, these inflammatory markers significantly decreased. In comparison, Se@Tri-PLNs gave rise to a remarkable downregulation toward the inflammatory factors. This may be attributable to the sensitization of Se to Tri that cooperatively inhibits the release of pro-inflammatory cytokines. Additional evidence comes from the thymus index and spleen index that mirror the status of the immune function [43]. It was found that the thymus index and spleen index of the DSS-induced group were significantly lower than that of the control group (Table 3), signifying the immune function of mice being inhibited. For the treatment group, both indexes escalated to varying degrees, wherein Se@Tri-PLNs demonstrated an optimal immune boosting effect. A histopathological check further validated that Tri-PLNs and Se@Tri-PLNs possessed excellent anti-UC activities, even at a considerably low dose (1 mg/kg) (Figure 11I). Compared with Tri-PLNs, the anti-UC efficacy of Se@Tri-PLNs was more prominent, which markedly reduced the lymphocytic infiltration of the mucosal epithelium and the severity of UC.

Se@Tri-PLNs not only have advantages in cellular uptake and transport, but also show better in vivo anti-UC action. The synergy between Se and Tri in inflammation resolution has been clearly documented. In fact, enteritis correlates with the progression of inflammation and oxidative stress. As a promising phytomedicine for IBD, Tri can act on the disease target by modulating oxidative stress, inflammatory cytokines, and intestinal homeostasis [44], suppressing the RIP3/MLIC necroptosis pathway [45] and maintaining immune balance by regulating gut microbiota [9]. Moreover, Se has been proven to be qualified with direct immunomodulatory and anti-inflammatory properties as an oxidative stress alleviator [46]. Therefore, selenization contributed substantially to the enhancement of nanoscaled Tri against UC.

## 4. Conclusions

In this work, we developed selenized polymer-lipid hybrid nanoparticles for oral delivery of Tri in an attempt to potentiate its anti-enteritis efficacy. We successfully prepared Se@Tri-PLNs through the solvent diffusion combined with in situ reduction technology. The resultant nanomedicine demonstrated numerous advantages in stability, drug release, cellular uptake, bioavailability, and anti-UC activity. Se@Tri-PLNs effectively prevented the progression of UC, lowered the levels of inflammatory factors in vivo, and alleviated DSS-associated injury to the colon compared with free Tri and Tri-PLNs. Ameliorative cellular uptake and pharmacokinetics were responsible for enhanced anti-enteritis activity. Se, as a sensitizer, further strengthened the curative effect of Tri. These findings provide a formulation solution to integrating water-insoluble API and Se into clinically applicable medications for the management of IBD. Selenized PLNs may serve as a promising nanotechnology platform for the oral delivery of anti-inflammatory agents to treat IBD.

## Figures and Tables

**Table 1:** Concentration and function of physiological inhibitors used in the cellular trafficking study.

Inhibitor	Concentration	Function
Hypertonic sucrose	0.5 M	Nonspecific inhibition to clathrin-mediated endocytosis
Chlorpromazine	25 μM	Specific inhibition to clathrin-mediated endocytosis
Simvastatin	25 μM	Nonspecific inhibition to caveolin-mediated endocytosis
Genistein	25 μM	Specific inhibition to caveolin-mediated endocytosis

**Table 2:** Main pharmacokinetic parameters after oral administration of Tri suspensions, Tri-PLNs and Se@Tri-PLNs in rats at a dose of 40 mg/kg.

PK Parameters	Tri Suspensions	Tri-PLNs	Se@Tri-PLNs
*C*_max_ (ng/mL)	293.12 ± 37.42	298.81 ± 22.50	237.50 ± 26.82 **
*T*_max_ (h)	4.00 ± 0.00	6.00 ± 2.00	7.33 ± 1.15 *
*AUC*_0–∞_ (ng/mL·h)	2186.46 ± 202.22	6130.28 ± 483.05 **	8675.95 ± 322.14 **
*t*_1/2_ (h)	5.05 ± 1.46	13.76 ± 1.58 **	13.77 ± 2.35 **

Data expressed as mean ± SD (*n* = 5). ANOVA, * *p* < 0.05, ** *p* < 0.01 compared with Tri suspensions.

**Table 3:** Thymus index and spleen index (*n* = 5, mean ± SD).

Group	Thymus Index	Spleen Index
Control	0.1769 ± 0.0155	0.4731 ± 0.0618
DSS	0.1187 ± 0.0126	0.2508 ± 0.0350
DSS + Tri	0.1177 ± 0.0087	0.2822 ± 0.0260
DSS + Tri-PLNs	0.1497 ± 0.0384	0.3681 ± 0.0350
DSS + Se@Tri-PLNs	0.1631 ± 0.0060	0.4131 ± 0.0536

## Data Availability

Data available on request from the corresponding author.
